# Cerebellar and subcortical interplay in cognitive dysmetria: functional network signatures associate with symptom and trait assessments across schizophrenia, bipolar II, and ADHD patients

**DOI:** 10.1007/s11682-025-01006-9

**Published:** 2025-04-23

**Authors:** Stacy N. Hudgins, Adrian Curtin, Joseph Tracy, Hasan Ayaz

**Affiliations:** 1 School of Biomedical Engineering, Science, and Health Systems, Drexel University, Philadelphia, PA USA; 2 Department of Neurology, Thomas Jefferson University, Philadelphia, PA USA; 3 Department of Psychological and Brain Sciences, College of Arts and Sciences, Drexel University, Philadelphia, PA USA; 4 A.J. Drexel Autism Institute, Drexel University, Philadelphia, PA USA; 5 Drexel Solutions Institute, Drexel University, Philadelphia, PA USA; 6 Center for Injury Research and Prevention, Children’s Hospital of Philadelphia, Philadelphia, PA USA

**Keywords:** Cognitive dysmetria, Cerebellum, Subcortex, Functional connectivity, Functional magnetic resonance imaging (fMRI)

## Abstract

Cognitive dysmetria suggests a disorganization of cognitive processes, particularly in relation to the cerebellum’s role in coordinating thoughts and actions. This phenomenon has been extensively studied in various psychiatric disorders, including schizophrenia (SCHZ), bipolar disorder II (BIPOL), and attention-deficit/hyperactivity disorder (ADHD). Understanding the relationship between cognitive dysmetria and functional connectivity in these disorders would reveal significant insights into their neurobiological underpinnings. This study explores how distinct and similar functional network connectivity (FNC) patterns between brain regions are associated with clinical symptoms and trait assessments across SCHZ, BIPOL, and ADHD patients by examining both working memory and task-free conditions compared to healthy volunteers (HC). Leveraging an open-source fMRI dataset from the UCLA Consortium for Neuropsychiatric Phenomics, we analyzed FNC patterns across 115 default mode and salience network regions, including cortical, subcortical, and cerebellar regions of interest in 135 participants (39 HC, 27 SCHZ patients, 38 BIPOL patients, and 31 ADHD patients). Abnormal FNC patterns compared to HC were localized to the cerebellar, thalamic, striatal, hippocampal, medial prefrontal and anterior insular cortices. Post-hoc multiple comparison analysis showed abnormal network connectivity predominantly in SCHZ and ADHD patients during rest, while the task condition demonstrated differential effects across all three disorders. Statistical analysis using a factor-by-covariance approach (GLM MANCOVA) suggested that regional functional connectivity was associated with select symptoms and traits pointing to neural signatures underlying psychiatric conditions. Our study suggests that examining and harnessing dysfunctional relationships in subcortical and cerebellar regions could provide a new perspective on the neurobiological basis of psychoses and help improve available treatment strategies.

**Supplementary Information:**

The online version contains supplementary material available at 10.1007/s11682-025-01006-9.

## Highlights

Cognitive dysmetria is characterized by disordered cortico-subcortico-cerebellar communication.

Cortico-subcortico-cerebellar regions show distinct patterns of engagement across SCHZ, BIPOL, and ADHD during both task and task-free conditions.

Functional connectivity imbalance in cortico-subcortico-cerebellar regions in task-free condition differs predominantly in Schizophrenia and ADHD patients.

Functional connectivity imbalances are present in all three disorders during the working memory task condition.

FNC findings related to select symptoms and traits reveal neural signatures underlying specific psychiatric conditions beyond diagnostic categories.

**Supplementary Information:**

The online version contains supplementary material available at 10.1007/s11682-025-01006-9.

## Introduction

Cortico-subcortico-cerebellar networks form crucial neural pathway connecting the cerebral cortex, subcortical structures, and cerebellum enabling integrated communication between regions responsible for motor coordination, sensory processing, and higher cognitive function (Cao & Cannon, 2019; Ha et al., 2023). Cognitive dysmetria proposes that the disorganization of cognition and behavior observed in psychiatric disorders can be explained in part by failures of the cerebellar network’s coordination of thoughts and actions (Cao & Cannon, 2019; Schmahmann et al., 2019). Research on schizophrenia (SCHZ) has revealed dysfunctional connectivity between the cerebellum and subcortex, highlighting the cerebellar role in impaired nonmotor cognitive function (Gong et al., 2019; Hamoda et al., 2019; Phillips et al., 2015). Supporting the importance of cognitive dysmetria, additional whole-brain MRI studies have revealed common structural and functional abnormalities involving the prefrontal cortex, insular cortex, thalamus, striatum, and cerebellum (Bernard & Mittal, 2015; Habas, 2021; Matsuo et al., 2013; Pinheiro et al., 2021; Ramsay, 2019; Wheeler & Voineskos, 2014). Studies have robustly detected hyperconnectivity in cerebellar-thalamic-cortical (CTC) circuitry across SCHZ patients in ways which associate with their symptoms (Cao et al., 2018). Recent evidence suggests that these changes may extend beyond SCHZ, with commonalities found in bipolar disorder II (BIPOL), and attention-deficit/hyperactivity disorder (ADHD) (Gong et al., 2020; Phillips et al., 2015; Pinheiro et al., 2021; Ramsay, 2019; Schmahmann et al., 2019; Shinn et al., 2017; Sorella et al., 2019). Additionally, BIPOL and SCHZ patients shared altered functional connectivity (FC) patterns within in the striatum, thalamus, hippocampus, amygdala, cerebellum and prefrontal cortex that associate with disruptions in emotional regulation, cognitive impairment, and working memory (Anticevic et al., 2014; Birur et al., 2017; Dimick et al., 2021; Du et al., 2018; Gong et al., 2020; Shinn et al., 2017; Sorella et al., 2019; Strakowski et al., 2005; Swann et al., 2009). These shared neural circuit abnormalities across psychiatric disorders underscores the transdiagnostic relevance of cognitive dysmetria, highlighting a need for comprehensive approaches to measure and characterize its neurobiological signatures.

Task-based and task-free functional connectivity approaches offer complementary advantages for understanding cognitive dysmetria. While task-based connectivity reveals how cognitive demands dynamically influence neural interactions, task-free connectivity highlights the intrinsic neural architecture supporting cognitive processes. In healthy brain function, a precise subcortical FC interplay between prefrontal and insular cortical loops prominently associates with specific cerebellar regions (crus1-2) during working memory, adaptive control, and cognitive processes (Habas, 2021; Schmahmann et al., 2019; Stoodley et al., 2016; Stoodley & Schmahmann, 2018). When this delicate interplay is interrupted, impaired regional coactivation during cognitive control can reveal distinctive intrinsic connectivity patterns that appear to be affected across multiple psychiatric disorders. Task-free resting state studies have further demonstrated that these abnormal connectivity patterns persist independently of task demands, suggesting they represent fundamental network alterations rather than task-specific deficits (Henseler et al., 2010). By associating specific FC disruptions with psychiatric symptoms and traits, researchers could improve the transdiagnostic prediction accuracy through the identification of shared impaired cognitive processes (Cole et al., 2021). This approach is particularly relevant because working memory impairments are robustly linked to the symptoms in SCHZ, BIPOL, and ADHD patients (Bernard & Mittal, 2015; Matsuo et al., 2013; Rubia, 2018; Tian et al., 2020a, b). Individuals with any of these three disorders frequently exhibit similar impairments in working memory tasks, which in turn can exacerbate symptoms of inattention and impulsivity (Ghosh et al., 2021). These cognitive deficits are thought to stem from dysfunctional interplay in the prefrontal cortex, subcortex and cerebellum, highlighting a commonality in declarative memory-related challenges across these disorders(Karlsgodt et al., 2010). Together, examining both task-based and task-free connectivity provides a more complete picture of the brain’s functional architecture and its adaptability to varying cognitive demands, offering new avenues for understanding psychiatric disorders from a network perspective.

Utilizing the functional neuroimaging datasets from the UCLA Consortium for Neuropsychiatric Phenomics (CNP) of working memory task and task-free conditions, we examine functional patterns and symptomatic features of cognitive dysmetria across SCHZ, ADHD, and BIPOL disorders compared to HC. Differences in functional neuroimaging responses between the cerebellum and subcortex may be attributable to the symptoms and traits underlying the psychiatric spectrum or to the experimental conditions (task-free or task-based) (Cole et al., 2021). By revealing specific associations between disrupted regional coactivation patterns and clinical symptom profiles, our findings enhance the transdiagnostic understanding of these disorders and point toward neural targets for intervention. This approach to examining cognitive dysmetria through dysfunction in cortico-subcortico-cerebellar networks not only offers new perspectives on the neurobiological basis of psychoses but also may inform more targeted therapeutic strategies that address shared cognitive mechanism across diagnostic boundaries.

## Methods

### Participants

Subjects were selected from an open access neuroimaging dataset from the UCLA CNP, consisting of135 adult male and female subjects between the ages of 21 and 50 years: 39 healthy volunteers (HC), 27 SCHZ, 38 BIPOL, and 31 ADHD subjects (Gorgolewski et al., 2017; Poldrack et al., 2016). Subjects from the CNP were excluded where known functional scan artifacts exist. Table 1 summarizes the demographic and clinical assessment group descriptive statistics for included participants. Primary diagnoses followed the DSM-IV TR criteria (Table 2A) through the Structured Clinical Interview for DSM-IV(American Psychiatric Association, 2000) and were complemented by the Adult ADHD Interview to characterize a history of ADHD in adults (Kaufman et al., 2000). The CNP cohort authors provided comorbidities whereby 81% of the patients had at least one comorbidity (Table 2B) and indicated a history of medicated treatment. Validated clinical assessments such as the Hopkins Symptom Checklist (HSCL), the Dickman Impulsivity Scales (DIS), Chapman Scales for Anhedonia (Chapman), Adult ADHD Self-Report Scale (ASRS), and Trait Scale for BIPOL Risk measured symptoms and traits. From the numerous assessments available in the dataset, we selected these specific measures because they included HC and were sensitive to declarative encoding and retrieval memory tasks (Ayres et al., 2021; Cools et al., 2007; Fortunato-Tavares et al., 2015; Landau et al., 2012) (further discussion can be found in the Supplemental section).

**Table 1:** The demographic and clinical data for the participants

		HC	SCHZ	BIPOL	ADHD	Statistics	
		Mean *±* SD	Mean *±* SD	Mean *±* SD	Mean *±* SD	F-Stat	*p* value
Sample Size (female/male)		39 (16/23)	27 (6/21)	38 (17/21)	31 (14/17)		
Age (years)		32.7 *±* 9.3	36.4 *±* 9.3	34.7 *±* 8.8	31.1 *±* 9.4		
*Post-hoc multiple pair-wise comparisons (p < 0.05 significance * *** h*** *: versus HC, * *** s*** *: versus SCHZ, * *** b*** *: versus BIPOL, and * ***a*** *: ADHD)*							
**Symptoms**							
Adult Self-Report Scale v1.1 Screener (ASRS)		9.3 *±* 2.6	10.4 *±* 4.3	13.1 *±* 5.2^h^	15.8 *±* 3.9^h, s^	16.88	< 0.001
Hopkins Symptom Checklist (HSCL)	Interpersonal Sensitivity	0.4 *±* 0.4	1.1 *±* 0.7^h^	1.1 *±* 0.8^h, a^	0.7 *±* 0.5	11.44	< 0.001
	Somatization	0.2 *±* 0.2	0.7 *±* 0.5^h, a^	0.7 *±* 0.7^h^	0.3 *±* 0.2	10	< 0.001
	Anxiety	0.3 *±* 0.3	0.9 *±* 0.7^h, a^	0.7 *±* 0.7^h^	0.4 *±* 0.4	9.94	< 0.001
	Obsessive-Compulsive	0.5 *±* 0.4	1.2 *±* 0.6^h^	1.2 *±* 0.8^h^	1.2 *±* 0.8^h^	10.32	< 0.001
	Depression	0.4 *±* 0.4	0.9 *±* 0.7^h^	1.0 *±* 0.6^h^	0.6 *±* 0.4	7.76	< 0.001
	Global Severity	0.4 *±* 0.3	0.9 *±* 0.5^h^	0.9 *±* 0.6^h^	0.6 *±* 0.4^h^	12.52	< 0.001
**Traits**							
Dickman Functional and Dysfunctional Impulsivity Scale	Functional Total	6.5 *±* 2.6	5.5 *±* 2.5	5.8 *±* 3.0	6.6 *±* 3.0	1.08	0.36
	Functional Positive	3.2 *±* 1.8	3.2 *±* 1.8	3.3 *±* 1.8	3.4 *±* 3.0	0.14	0.94
	Functional Negative	3.3 *±* 1.4	2.3 *±* 1.3^h^	2.5 *±* 1.5	3.2 *±* 1.7	3.94	0.01
	Dysfunctional Total	1.3 *±* 1.8	4.5 *±* 3.1^h^	6.2 *±* 4.3^h^	4.9 *±* 2.9^h^	16.59	< 0.001
	Dysfunctional Positive	0.8 *±* 1.8	3.4 *±* 5.2^h^	4.3 *±* 3.0^h^	3.6 *±* 2.3^h^	15.03	< 0.001
	Dysfunctional Negative	0.46 *±* 0.6	1.1 *±* 1.4	1.9 *±* 1.5^h^	1.3 *±* 1.2^h^	11.52	< 0.001
Chapman Scales	Physical Anhedonia	10.4 *±* 6.3	16.5 *±* 7.2^h^	16.6 *±* 9.1^h^	13.6 *±* 6.0	5.83	< 0.001
	Social Anhedonia	9.4 *±* 6.2	16.0 *±* 6.2^h^	16.0 *±* 7.1^h^	14.1 *±* 7.6^h^	7.57	< 0.001
Scale for Traits that Increase Risk for Bipolar II Disorder		10.7 *±* 4.8	15.4 *±* 5.6^h^	17.5 *±* 6.2^h^	14.6 *±* 3.6^h^	11.52	< 0.001

Abbreviations: HC: Healthy Controls; SCHZ: Schizophrenia; BIPOL: Bipolar II Disorder; ADHD: Attention Deficit Hyperactivity Disorder; F-stat: F-statistic one-way ANOVA for each symptom/trait assessment

**Table 2:** Primary patient diagnosis with associated comorbidities

**A) Primary diagnostic information for patient groups**	
**Diagnosis (DSM IV code)**	***N*** **(Participant Count)**
SCHZ, Schizophrenia Disorders (295.xx)	27
BIPOL, Mood Disorders, Most Recent Episode (296.40–70)	38
ADHD, Attention-Deficit and Disruptive Behavior Disorders (314.xx)	31
**B) Comorbid conditions present for patients.**	
**Diagnosis (DSM IV code)**	**N (Participant Count)**
Substance Dependence (303.90 -304.90)	160
Substance Abuse (305.00–305.90)	70
Mood and Anxiety-Related Disorders (296.xx, 300.xx, 309.28, 311, and 314.xx)	72
Other Comorbid Diagnoses (292.12 and 292.84)	2

Note: The “xx” in some codes represents additional digits used for more specific diagnoses within each category. The full DSM-IV contains hundreds of specific diagnostic codes, each with its own set of criteria

Abbreviations: SCHZ: schizophrenia, BIPOL: bipolar II, ADHD: attention deficit/hyperactivity disorder

Adapted from Poldrack, R., et al. A phenome-wide examination of neural and cognitive function. Sci Data 3, 160,110 (2016)

### Study design and procedures

Subjects attended two order-counterbalanced experimental sessions, one resting-state session and one cognitive session. During the resting-state session, subjects were relaxed with their eyes open during a 304-second fMRI scan. During the cognitive session, two scanning sessions were performed to assess declarative memory encoding and retrieval using paired-association memory (PAM) tasks (Poldrack et al., 2016). The encoding session (PEncode) consisted of 40 ‘memory’ trials and 24 ‘control’ trials (64 total), lasting 8 min per subject. During each memory trial, two words were presented adjacent to each other for 1s. Then, line drawings of two objects matching the words appeared above the words and were presented together for 3 more seconds (4s total). One object was traced in black and white, and the other was traced using a single color. The control trials consisted of scrambled stimuli pairs appearing for 2s, one black and white and one colored stimulus. After each trial, the subject indicated which side of the screen contained the colored object were instructed to remember the objects and their paired relationships.

The retrieval session consisted of 40 correct trials(PCorrect), 40 incorrect trials (PIncorrect), and 24 control trials (total 104) presented in a pseudorandom order and lasted 8.93 min per subject. For each retrieval memory trial, two adjacent objects were presented for 3s, after which the subjects were asked to rate their confidence that the two objects had been paired together during the encoding session. During correct trials, only originally paired objects were presented, whereas during incorrect trials, mismatched objects were presented. Control trials consisted of scrambled stimuli pairs appearing for 2s.

### Acquisition and processing

fMRI data were acquired on 3T Siemens Trio scanners using a T2-weighted echoplanar imaging(EPI) sequence following: 4 mm slice thickness, 34 slices, 2s TR, 30ms TE, 90° flip angle, 64 × 64matrix, 192 mm FOV, and oblique slice orientation. The encoding session comprised 242 TRs, 104(42.9%) considered active tasks. The retrieval session comprised 268 TRs, 156 (58.2%) considered to be active tasks. Further descriptions of the image technical validation and quality control methods used are provided by the CNP authors (Poldrack et al., 2016).

Functional images were realigned, unwarped, and slice-time corrected. Three functional-based parcellation schemes were used to define 45 ROIs within the cerebellum (Ren et al., 2019), 16 ROIs within the cerebral cortex (Schaefer et al., 2018), and 54 ROIs within the subcortex (Y. Tian et al., 2020a, b) (see Suppl Material). 115 ROIs (cortical = PFC [prefrontal cortex], insular, and angular; cerebellum = crusI-II, lobules VI, and VIIb; subcortical = hippocampus, amygdala, thalamus, and basal ganglia) were selected as seeds for between-source contrasts for both resting-state and generalized psychophysiological index (gPPI)(McLaren et al., 2012) analyses to assess interactions in predominantly subcortical and non-motor cerebellar regions. We limited seed cortical regions to those associated with executive and salience functioning. Functional data were segmented, normalized and coregistered to MNI space, as well as motion-corrected. Accepted data were then bandpass filtered and linear detrended with no spiking. Connectivity maps were generated for each subject in both task and task-free conditions. Detailed preprocessing, denoising, and within-subject seed-based and gPPI analysis methods are provided in the supplemental material.

### Data analysis

Group-level analyses were performed using General Linear Models (GLM) (Whitfield-Gabrieli & Nieto-Castanon, 2012). For each individual voxel a separate GLM was estimated with first-level connectivity measures as dependent variables (one independent sample per subject at rest and one measurement per task condition), and groups or other subject-level identifiers as independent variables. ROI-level hypotheses were evaluated using multivariate parametric statistics with random-effects across subjects and sample covariance estimation across multiple measurements. Inferences were performed at individual clusters (groups of contiguous voxels). Cluster-level inferences were based on parametric statistics from Gaussian Random Field theory (Worsley et al., 1996). Results were thresholded using a combination of a cluster-forming *p* < 0.001 voxel-level threshold, and a familywise corrected p-FDR < 0.05 cluster-size threshold (Chumbley et al., 2010).

For the task-free condition, FC differences across the subject groups were assessed via ANOVA interaction using a between-subject contrast (HC vs. SCHZ, BIPOL, and ADHD patients). For each task condition, FC differences across the subject groups were assessed using a 4 × 2 mixed ANOVA interaction using a between-subject contrast in the task-free condition and between-condition contrast (control vs. task condition). In both task-free and task conditions, hierarchical clustering of all ROI pairs was examined for within- and between-intrinsic subnetwork connectivity using ROI-to-ROI functional network connectivity clusters that survived multivariate parametric GLM testing (FNC; further details found in the supplemental material) (Jafri et al., 2008). Significant seed-target ROI pairs were subsequently identified within surviving FNC sets where the connection threshold was set at *p* < 0.05 p-FDR correction.

Using between-subject effects, we tested for interactions between significant pairwise ROIs (covariates) and symptom/trait assessments (factors). We conducted a GLM-based MANCOVA examining between-subjects effects where previously determined significant seed-target ROI pairs from FNC clusters covaried (Table 3: F-statistic shown for *p* < 0.05 significance). We used a full-factorial design in the task-free condition and factor-by-covariate design in the task conditions.

**Table 3:** Between-subject effects testing interactions between significant pairwise rois (covariates) and symptom/trait assessments (factors)

**Task-free**						
	**HSCL-OC**	**HSCL-Dep**	**HSCL-GS**	**Chap-PhysAnh**	**Chap-SocAnh**	**BipolarII Scale**
L-DA-PUT → L-Crus I				3.97 (*p* = 0.048)		
L-THA-DAm → R-PUT-DP				8.32 (*p* = 0.005)		
L-CAU-tail → R-pGP	4.37 (*p* = 0.039)	7.78 (*p* = 0.006)	5.09 (*p* = 0.026)			
L-THA-VAia → L-DefaultA-PFCm					7.49 (*p* = 0.007)	5.45 (*p* = 0.021)
**Encoding Task (PEncode)**						
	**DIS-FuncTot**	**DIS-FuncPos**	**DIS-DysFuncTot**	**DIS-DysFuncNeg**	**Chap-SocAnh**	**BipolarII Scale**
R-CAU-body → R-THA-VPl			4.19 (*p* = 0.043)	6.14 (*p* = 0.015)		
L-THA-VAia → L-HIP-head					4.26 (*p* = 0.041)	
R-THA-VAip → L-PUT-DP	4.22 (*p* = 0.042)	9.75 (*p* = 0.002)				5.97 (*p* = 0.016)
**Retention Task– Congruent (PCorrect)**						
	**DIS-FuncTot**	**DIS-FuncPos**	**Chap-PhysAnh**			
R-VIIb -Crus II → R-Crus II	5.06 (*p* = 0.026)	6.39 (*p* = 0.013)	5.14 (*p* = 0.025)			
**Retention Task– Incongruent (PIncorrect)**						
			**ASRS**			
L-HIP-head → L-THA-DAm			4.11 (*p* = 0.045)			

*Full-Factorial (task-free) and Factor-by-Covariate (task) Generalized Linear Model MANOVA Tests of Between-Subjects Effects where previously determined ROIs from survived FNC clusters covary: F-Statistic (p-value) shown for p < 0.05 significance*

Abbreviations: ASRS: Adult Self-Report Scale v1.1; HSCL: Hopkins Symptom Checklist; DIS: Dickman functional and dysfunctional impulsivity scale; Chap: Chapman Scales for social anhedonia and physical anhedonia; L: Left; R: Right; DA: dorsal anterior; PUT: putamen; Crus: cerebellar crus; THA: thalamus; DAm: dorsal anterior medial; DP: dorsal posterior; CAU: caudate; pGP: posterior globus pallidus; VAia: ventral anterior inferior anterior; VAip: ventral anterior inferior posterior; DefaultA-PFCm: medial prefrontal cortex in the default mode network; HIP: hippocampus; VIIb: cerebellar lobule VIIb; df: degrees of freedom; F-stat: F-statistic from comparing multiple independent variables

## Results

### Participant demography, symptom and trait assessments

Demographic data, clinically assessed symptoms, and traits are presented in Table 1. No statistical differences in demographics were observed between participant groups (*p* > 0.05). One-way ANOVAs conducted for each symptom and trait assessment measure (F[3,134] > 3.9, *p* > 0.05). Post-hoc multiple pair-wise comparisons between participant groups were conducted for each measure (Table 1: *p* < 0.05 significance h: versus HC, s: versus SCHZ, b: versus BIPOL, and a: ADHD).

### Resting-state FNC patterns

**Fig. 1:**
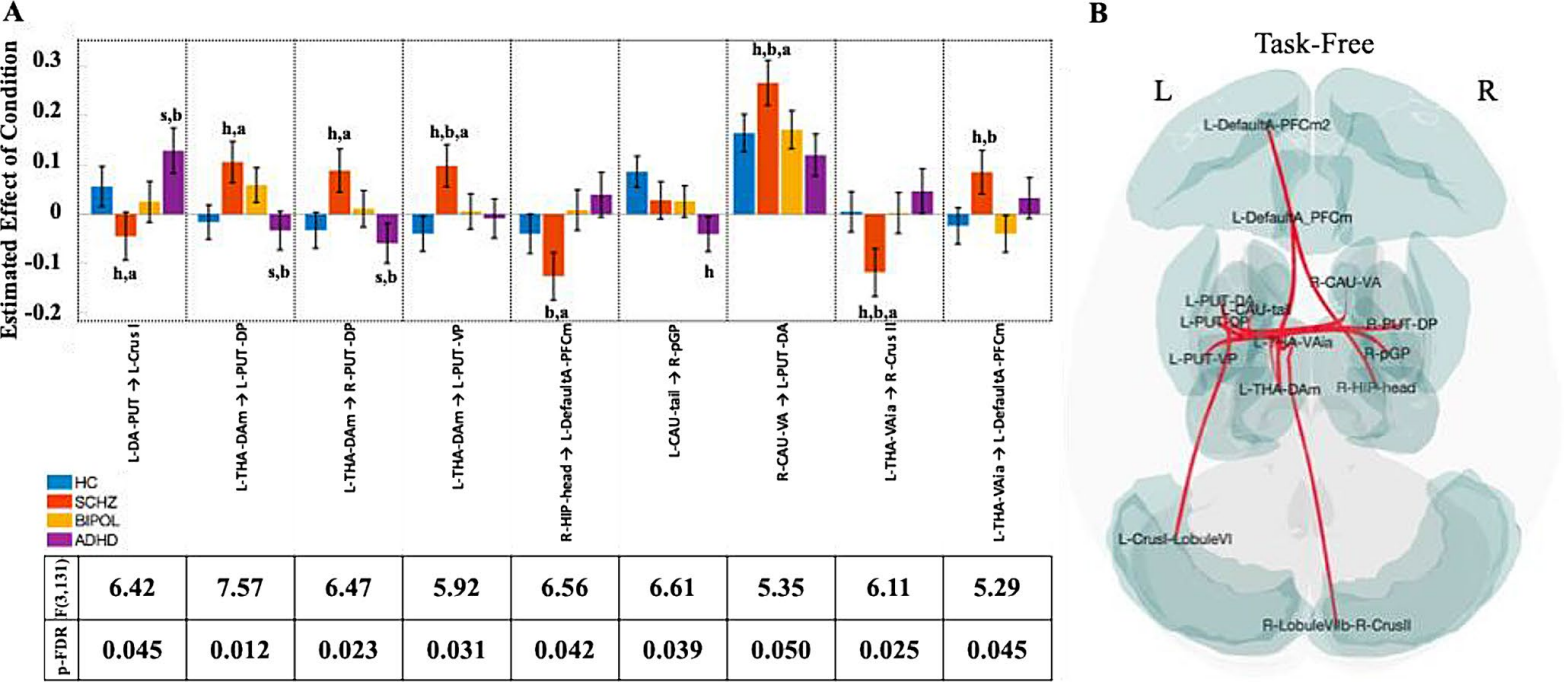
Task-free functional network connectivity (FNC) analysis in HC, SCHZ, BIPOL, and ADHD participants. Cluster-based inference connections and cluster thresholds for 231 clusters. ANOVA interaction, between-subject contrast SCHZ patients compared to healthy controls (SCHZ > HC), bipolar (BIPOL > HC), and ADHD (ADHD > HC) patients. **A**: Bar graphs represent the between-subject estimated effect of task-free condition within each significant ROI pair with corresponding F-statistic and p-FDR value shown. Notations in the graph represent post-hoc Bonferroni multiple comparison tests conducted on ROI pair r-transformed Z statistic (*p* < 0.05): h) versus HC, s) versus SCHZ, b) versus BIPOL, and a) ADHD. **B**: Overlaid reference ROIs used in the analysis (bilateral frontal and insular cortices, subcortical regions, and cerebellar crusI and crusII); seed-based ROI-to-ROI representation for significant ROIs found within significant FNC cluster sets (seed = regional parcel and ROI-to-ROI connection represents the F-statistic)

Multivariate parametric FNC testing compared resting-state group differences (between SCHZ, BIPOL, and ADHD patients, HC) and observed 9 significant differential regional pairs (Fig. 1: F[3,131] > 5.29, p-FDR < 0.05). Post-hoc Bonferroni multiple comparison tests on ROI pair r-transformed Z-statistics determined whether one or more patient groups predominated individual ROI pairs (Fig. 1A, *p* < 0.05 Bonferroni-corrected). When compared to HC, the SCHZ group significantly influenced 8 of the 9 significant ROI pairs However, the left caudate tail - right posterior globus pallidus pair was more significantly influenced in the ADHD patient group compared to HC. ROI pairs in BIPOL patients did not significantly differ from any other participant group.

### gPPI task FNC Patterns - Paired-Association memory (PAM)

**Fig. 2:**
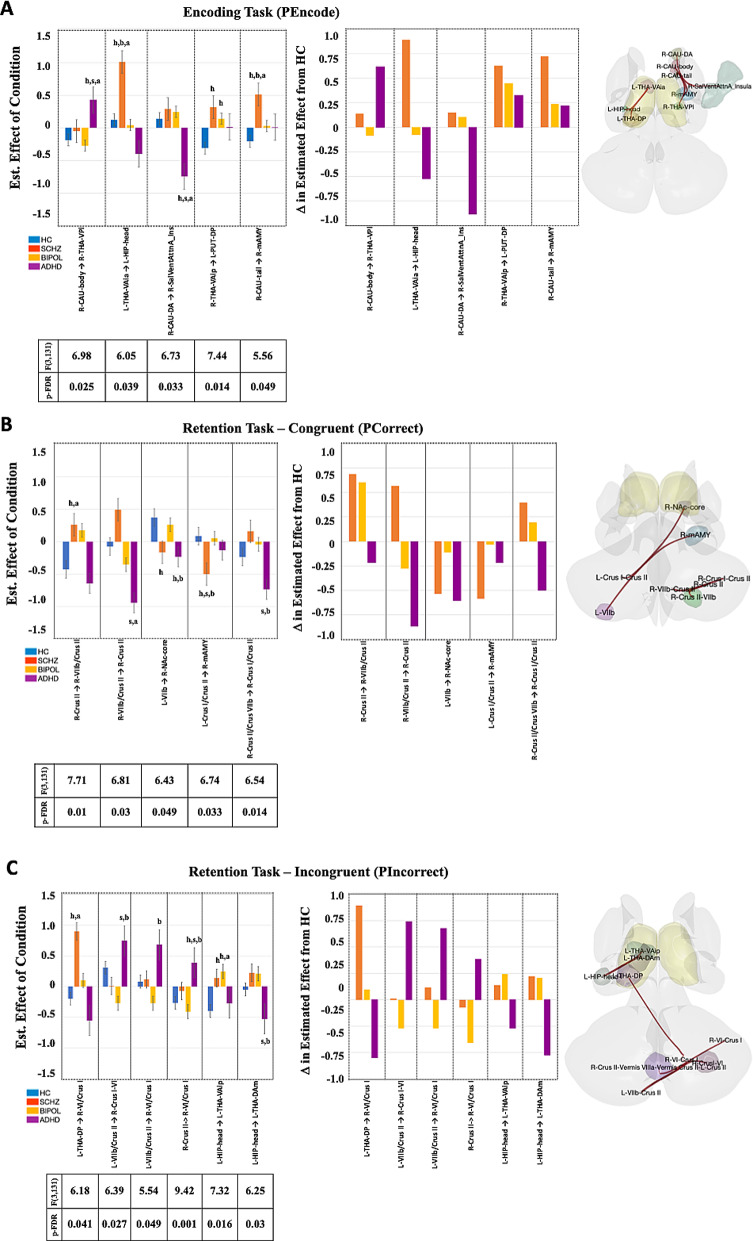
Functional network connectivity (FNC) sets of paired-association memory conditions using generalized psychophysiological indexes (gPPI) in HC, SCHZ, BIPOL, and ADHD participants. **A**: PAM encoding (PEncode). **B**: correct match during PAM retrieval (PCorrect); and **C**: incorrect match during PAM retrieval (PIncorrect). Cluster-based inference with cluster and connection thresholds at *p* < 0.05 was FDR-corrected (clusters: 185, 166, and 204). 4 × 2 mixed ANOVA interaction, between-subject contrast (HC, SCHZ, BIPOL, and ADHD) and within-subject contrast separately for each condition (**A**: PEncode > Control, **B**: PCorrect > Control, and **C**: PIncorrect > Control). For each condition **A**-**C**, the first bar graph represents the estimated effect of the condition between-subjects for each significant ROI pair with corresponding F-statistics and p-FDR values. Notations in the graphs represent post-hoc Bonferroni multiple comparison tests conducted on ROI pair r-transformed Z statistic, *p* < 0.05 significance: h) versus HC, s) versus SCHZ, b) versus BIPOL, and a) ADHD. The second bar graph represents the change (**D**) in estimated effect from HC. The neuroimage represents seed-based ROI-to-ROI anatomy of the significant ROIs identified (seed = regional parcel and ROI-to-ROI connection represents the F-statistic)

Following psychophysiological interaction (PPI) analyses, separate cluster-based multivariate parametric FNC tests using a 4 × 2 mixed ANOVA interaction design compared between-subject group differences between SCHZ patients, BIPOL patients, and ADHD patients from HC in each task condition. 185, 166, and 204 clusters were generated, respectively. However, only three to four FNC sets survived parametric testing (F[3,131] > 5.54, p-FDR < 0.05). Within these FNC sets for the PEncode between-conditions contrast (Fig. 2A, PEncode > Control), significant paired ROIs arose from the right dorsal anterior caudate, body, and tail as well as the bilateral ventral anterior thalamus. In the PCorrect between-conditions contrast (Fig. 2B, PCorrect > Control), significant cerebellar paired ROIs arose from right crus II, lobule VIIb and left crus I. For the PIncorrect condition (Fig. 2C, PIncorrect > Control), significant paired ROIs arose from the left hippocampal head, left posterior thalamus, and overlapping cerebellar vermis with bilateral crus II, left lobule VIIb, and left crus II.

Following post-hoc pairwise between-group multiple comparison tests on ROI pair r-transformed Z statistic, we determined between-group differences in each condition for each ROI pair (Fig. 2A-C: a versus HC, b versus SCHZ, c versus BIPOL, and d versus ADHD at *p* < 0.05 Bonferroni-corrected). The PEncode condition revealed unique ROI pair relationships predominantly for SCHZ, as well as for ADHD patients compared to all the other groups. Whereas FC relationships between the ventral anterior thalamus and dorsal posterior putamen were similarly significant in SCHZ and BIPOL. In the PCorrect condition compared to HC, three ROI pairs in SCHZ, four pairs in the ADHD group, and one pair in the BIPOL involved the cerebellum, nucleus accumbens core, and medial amygdala. Compared to HC in the PIncorrect condition, two significant ROI pairs in the SCHZ, one significant ROI pair in BIPOL, and one significant ROI pairs in ADHD involved the cerebellum, hippocampal head, as well as ventral anterior and dorsal thalamus were observed.

### Between-subject interactions between significant ROI pairs and symptom and trait assessments

#### Task-free conditions

No significance was found following a full-factorial GLM MANOVA test among the previously determined task-free ROI pairs across diagnosis, symptom and trait assessments: Wilke’s Λ > 0.82, F[14,108] < 1.67, *p* > 0.05, η^2^ < 0.178. Tests of normality and equal variance were not violated with sufficient power (> 0.80). For each ROI pair showing significant interactions below, matched clinical group differences from earlier FNC indications are referenced in parentheses.

Left-to-right caudopallidal connectivity demonstrated intersubject covariation with HSCL-measured compulsive, depressive, and global symptom dimensions (F[1,12] > 4.37, *p* < 0.039, η^2^ > 0.035; ADHD > HC in 3.2). Additionally, Striatocerebellar (dorsal putamen) (F[1,12] = 3.97, *p* = 0.048, η^2^ = 0.032), as well as left-to-right thalamostriatal (dorsomedial) (F[1,12] = 8.32, *p* = 0.005, η^2^ = 0.064; SCHZ > HC in 3.2) connectivity patterns covaried with physical anhedonia severity (Chapman PAS), particularly involving cerebellar crus I and the posterior putaminal nodes. Lastly, Left ventral anterior thalamic connectivity with default mode prefrontal regions demonstrated significant associations with Chapman social anhedonia and bipolar II risk metrics (F[1,12] > 5.45, *p* < 0.021, η^2^ > 0.043; SCHZ > HC in 3.2).

#### Task conditions

Separate factor-by-covariate GLM MANCOVAs, conducted for each task condition among the previously determined ROI pairs, revealed no significant effects across diagnosis, symptom, and trait assessments: Wilke’s Λ > 0.84, F[14,115] < 1.56, *p* > 0.05, η^2^ < 0.160. Tests of normality and equal variance were not violated with sufficient power (> 0.80).

Following between-subject effects tests in the PEncode condition, thalamostriatal connectivity (right VA ↔ left posterior putamen) demonstrated dual associations with trait impulsivity (DIS total/positive) and bipolar II susceptibility markers (F[1,8] > 5.97, *p* < 0.042, η^2^ > 0.045; ADHD/SCHZ > HC in 3.3-PEncode). Right associative striatum (caudate body) connectivity with sensorimotor thalamus (VPL) covaried with DIS-measured impulsive avoidance (negative subscale) and global dysfunction (F[1,8] > 4.19, *p* < 0.043, η^2^ > 0.032; ADHD > HC in 3.3-PEncode). Lastly, social anhedonia severity (Chapman SAS) covaried with functional coupling between the left ventral anterior thalamus and hippocampal head, suggesting impaired limbic-thalamic integration (F[1,8] = 4.26, *p* = 0.041, η^2^ = 0.033; ADHD > HC in 3.3-PIncorrect).

Following between-subject effects tests in both PAM retrieval conditions separately, physical anhedonia (Chapman PAS) and impulsivity traits (DIS total/positive) covaried with intersubject differences in the cerebello-cognitive network (right crus II) connectivity in the PCorrect condition (F[1,8] > 5.06, *p* < 0.026, η^2^ > 0.039; ADHD > HC in 3.3-PCorrect). In the PIncorrect condition, ADHD symptom severity (ASRS) covaried with functional coupling between the left hippocampal head and dorsomedial thalamus (F[1,8] = 4.11, *p* = 0.045, η^2^ > 0.032; ADHD > HC in 3.3-PIncorrect).

## Discussion

Previous research has emphasized that subcortical structures are critical in the neuropathology of various psychiatric disorders, including SCHZ and BIPOL; however, the involvement of cerebellar regions and the functional interplay between subcortical regions in these disorders remains underexplored (Alves et al., 2019; Anticevic et al., 2014; Ferri et al., 2018; Hwang et al., 2022; Matsuo et al., 2013; Tu et al., 2020). Using FC methods, our analyses revealed state-dependent abnormalities between specific subcortical and cerebellar regions across SCHZ, BIPOL, and ADHD patients. Furthermore, we demonstrate that state-dependent functional neuroimaging responses between the cerebellum and subcortex correlate with specific psychiatric symptom and trait assessments. By demonstrating the linkage between impaired coactivation during cognitive control processes and underlying psychiatric symptoms and traits, our study provides a new perspective on the neurobiological basis of cognitive dysmetria that could inform the development of novel treatment strategies.

In the context of cognitive dysmetria, abnormal regional interactions occur during cognitive processing, with disrupted connectivity between the cerebellum with the prefrontal cortex and the subcortex representing a key example of such abnormalities (Gong et al., 2019; Hamoda et al., 2019). Our reported abnormal task-free findings between subcortical and cerebellar regions, predominantly in the SCHZ group, were consistent with previously reported findings (Ferri et al., 2018; Habas, 2021; Hwang et al., 2022; Scangos et al., 2023; Schmahmann et al., 2019; Stoodley & Schmahmann, 2018). Furthermore, our declarative memory task findings emphasize the state-dependent abnormal subcortical and cerebellar relationships across a psychiatric spectrum. Although these observations suggest these regional patterns associate with cognitive dysmetria (Chen et al., 2019), we observed contrasting effects predominantly between SCHZ and ADHD in network organization. Specifically, SCHZ patients showed hyperconnectivity between the ventral anterior thalamus, hippocampus (PEncode), cerebello-cognitive network (PCorrect), as well as the dorsal posterior (and anterior) thalamus, cerebellar crus-I, and hippocampus (PIncorrect), while ADHD patients demonstrated distinct dysconnectivity patterns in these same regions. Connectivity differences between subcortical and cerebellar regions in ADHD, may reflect different functional architecture than in SCHZ as a possible compensatory mechanisms or disruptions particularly in the limbic-attentional integration (Friston et al., 1997; Rolls et al., 2021). This suggests that while both disorders exhibit cerebellar involvement, the functional implications reflect the unique pathophysiological processes underlying each condition.

Overall, 81% of our subjects had at least one comorbidity (e.g. anxiety, impulsivity, depression). We suspected a likelihood for the underlying FC to overlap in both task and task-free conditions and hypothesized that a state-dependent influence of the subcortical and cerebellar interplay could associate with clinical assessments (Hwang et al., 2022; Tu et al., 2020). In task-free conditions, ADHD was associated with obsessive-compulsion, depression, and global severity (HSCL) measures via the paired caudate and posterior globus pallidum, and SCHZ was associated with physical anhedonia (Chapman) via the paired dorsal anterior thalamus and dorsal posterior putamen. Whereas in the PEncode condition, SCHZ and BIPOL functional impulsivity (DIS) was associated with the ventral anterior thalamus and dorsal posterior putamen, yet ADHD dysfunctional impulsivity (DIS) was associated instead with the ventral posterior thalamus and caudate connectivity. These differential connectivity-symptom associations across conditions are consistent with consistent with prior research showing that cognitive task demands modulate the cortico-subcortico-cerebellar coordination (Eckert et al., 2009) with distinct disruption patterns of disruption in psychiatric populations (DeSerisy et al., 2020; Jimenez et al., 2016; Rubia, 2018; Vieira et al., 2021; Wen et al., 2021). Given the modest effect sizes in our analysis, it is also plausible that declarative memory tasks may have limitations for distinguishing symptom-connectivity interactions between and other disorders (SCHZ and ADHA). Nevertheless, we did identify a significant interaction between the trait risk scale for BIPOL and functional connectivity between with the right ventral anterior thalamus and left dorsal putamen (PEncode), supporting the value of our approach. Together, state-dependent conditions in concert with clinical assessments provide compelling evidence for the central role of cortico-subcortical-cerebral network interactions in the neurobiological architecture underlying psychiatric spectrum disorders.

Our findings have several methodological limitations and alternative conceptualizations. While our study focused primarily on the interplay of the subcortical and non-motor regions of the cerebellum, we limited cortical regions to include only the prefrontal and anterior insular regions. This poses a methodological limitation by not including other significantly shared cortical regions engaged in demanding working memory tasks. This limitation presents is an opportunity for further investigation in the context of cortical-subcortical-cerebellar interplay. Second, patients from the CNP cohort were reported to be exposed to or receiving medicated treatment during the time of the study. Previous studies have demonstrated that antipsychotics, anti-depressants and mood stabilizers affect brain FCs; therefore, the use of treatment medications in our patient cohort may be confounding our findings in this study (Tu et al., 2020). Despite these limitations, our results provide valuable insights into the functional connectivity patterns across psychiatric disorders that align with prior research on cognitive dysmetria.

## Conclusion

In conclusion, our study establishes that interactions between subcortical and cerebellar regions exhibit state-dependent patterns in patients with SCHZ, BIPOL, and ADHD that associate with their symptoms and trait assessments. These observations are consistent with findings on the integration of the basal forebrain, cerebellar subregions, striatum, and the thalamus, where altered connectivity was observed in SCHZ and ADHD (Alves et al., 2019). Our findings compel further investigation into the transdiagnostic potential of cognitive control imbalances mediated by specific subcortical and nonmotor regions of the cerebellum. This evidence supports the cognitive dysmetria framework and suggests that investigating and potentially modulating aberrant connectivity patterns in subcortical and cerebellar regions may offer novel insights into the neurobiological underpinnings of various psychiatric disorders. This approach could provide a fresh perspective on the shared neural substrates across the psychiatric spectrum and potentially inform the development of more targeted and effective treatment strategies.

## Electronic supplementary material

Below is the link to the electronic supplementary material.

Supplementary Material 1

## Data Availability

No datasets were generated or analysed during the current study.
