# Supplementary material for: Cerebellar and subcortical interplay in cognitive dysmetria: functional network signatures associate with symptom and trait assessments across schizophrenia, bipolar II, and ADHD patients
Source: Brain Imaging Behav. 2025 Apr 23;19(3):759–70. doi: 10.1007/s11682-025-01006-9 (PMC12198319; doi:10.1007/s11682-025-01006-9)
Supplement: Supplementary file 1 — Supplementary Material 1 [file 11682_2025_1006_MOESM1_ESM.docx]

# Cerebellar and Subcortical Interplay in Cognitive Dysmetria: Functional Network Signatures Associate with Symptom and Trait Assessments across Schizophrenia, Bipolar II, and ADHD Patients

Stacy N. Hudgins ^1^, Adrian Curtin, PhD ^1^, Joseph Tracy, PhD ^2^, and Hasan Ayaz, PhD ^1,3-6^

1 School of Biomedical Engineering, Science, and Health Systems, Drexel University, Philadelphia, PA USA

2 Department of Neurology, Thomas Jefferson University, Philadelphia, PA, USA

3 Department of Psychological and Brain Sciences, College of Arts and Sciences, Drexel University, Philadelphia, PA USA

4 A.J. Drexel Autism Institute, Drexel University, Philadelphia, PA, USA

5 Drexel Solutions Institute, Drexel University, Philadelphia, PA USA

6 Center for Injury Research and Prevention, Children’s Hospital of Philadelphia, Philadelphia, PA USA

**Supplementary Section**

**Detailed Preprocessing Pipeline and Analysis Methods**

Results included in this manuscript come from analyses performed using CONN ([Whitfield-Gabrieli & Nieto-Castanon, 2012](#_ENREF_28)) (RRID:SCR_009550) release 21.a and SPM ([Penny, 2011](#_ENREF_20)) (RRID:SCR_007037) release 12.7771.

**Preprocessing**: Functional and anatomical data were preprocessed using a flexible preprocessing pipeline ([Whitfield-Gabrieli & Nieto-Castanon, 2012](#_ENREF_28)) including realignment with correction of susceptibility distortion interactions, slice timing correction, outlier detection, direct segmentation and MNI-space normalization, and smoothing. Functional data were realigned using SPM realign & unwarp procedure ([Andersson et al., 2001](#_ENREF_2)), where all scans were coregistered to a reference image (first scan of the first session) using a least squares approach and a 6 parameter (rigid body) transformation ([Friston et al., 2004](#_ENREF_12)), and resampled using b-spline interpolation to correct for motion and magnetic susceptibility interactions. Temporal misalignment between different slices of the functional data was corrected following SPM slice-timing correction (STC) procedure ([Sladky et al., 2011](#_ENREF_25)), using sinc temporal interpolation to resample each slice BOLD timeseries to a common mid-acquisition time. Potential outlier scans were identified using ART ([Morfini et al., 2023](#_ENREF_19)) as acquisitions with framewise displacement above 0.5 mm or global BOLD signal changes above 3 standard deviations ([Morfini et al., 2023](#_ENREF_19); [Power et al., 2014](#_ENREF_21)), and a reference BOLD image was computed for each subject by averaging all scans excluding outliers. Functional and anatomical data were normalized into standard MNI space, segmented into grey matter, white matter, and CSF tissue classes, and resampled to 2 mm isotropic voxels following a direct normalization procedure ([Calhoun et al., 2017](#_ENREF_6); [Morfini et al., 2023](#_ENREF_19)) using SPM unified segmentation and normalization algorithm ([Ashburner, 2007](#_ENREF_3); [Ashburner & Friston, 2005](#_ENREF_4)) with the default IXI-549 tissue probability map template. Last, functional data were smoothed using spatial convolution with a Gaussian kernel of 6 mm full width half maximum (FWHM).

**Denoising**: In addition, functional data were denoised using a standard denoising pipeline ([Whitfield-Gabrieli & Nieto-Castanon, 2012](#_ENREF_28)) including the regression of potential confounding effects characterized by white matter timeseries (5 CompCor noise components), CSF timeseries (5 CompCor noise components), motion parameters and their first order derivatives (12 factors) ([Friston et al., 1996](#_ENREF_14)), outlier scans (below 59 factors) ([Power et al., 2014](#_ENREF_21)), session effects and their first order derivatives (2 factors), and linear trends (2 factors) within each functional run, followed by bandpass frequency filtering of the BOLD timeseries ([Hallquist et al., 2013](#_ENREF_15)) between 0.008 Hz and 0.09 Hz. CompCor ([Behzadi et al., 2007](#_ENREF_5); [Chai et al., 2012](#_ENREF_7)) noise components within white matter and CSF were estimated by computing the average BOLD signal as well as the largest principal components orthogonal to the BOLD average, motion parameters, and outlier scans within each subject's eroded segmentation masks. From the number of noise terms included in this denoising strategy, the effective degrees of freedom of the BOLD signal after denoising were estimated to range from 60 to 79.4 (average 76) across all subjects ([Morfini et al., 2023](#_ENREF_19)).

**First-level seed-based analysis**: Seed-based connectivity maps (SBC) and ROI-to-ROI connectivity matrices (RRC) were estimated characterizing the patterns of functional connectivity with 115 ROIs. Functional connectivity strength was represented by Fisher-transformed bivariate correlation coefficients from a weighted general linear model (weighted-GLM ([Whitfield-Gabrieli & Nieto-Castanon, 2012](#_ENREF_28))), defined separately for each pair of seed and target areas, modeling the association between their BOLD signal timeseries. Individual scans were weighted by a boxcar signal characterizing each individual task or experimental condition convolved with an SPM canonical hemodynamic response function and rectified.

**First-level gPPI analysis**: Psychophysiological interaction analyses were used to study the changes in functional connectivity across Control, PEncode, PCorrect and PIncorrect conditions. Seed regions included 115 ROIs. Separately for each pair of seed and target areas, a generalized psychophysiological interaction model (gPPI ([Friston et al., 1997](#_ENREF_13); [McLaren et al., 2012](#_ENREF_18))) was defined with seed BOLD signals as physiological factors, boxcar signals characterizing each individual task condition convolved with an SPM canonical hemodynamic response function as psychological factors, and the product of the two as psychophysiological interaction terms. Functional connectivity changes across conditions were characterized by the multivariate regression coefficient of the psychophysiological interaction terms in each model.

**Group-level analyses** were performed using a General Linear Model (GLM ([Whitfield-Gabrieli & Nieto-Castanon, 2012](#_ENREF_28))). For each individual voxel a separate GLM was estimated, with first-level connectivity measures at this voxel as dependent variables (one independent sample per subject and one measurement per task or experimental condition, if applicable), and groups or other subject-level identifiers as independent variables. Voxel-level hypotheses were evaluated using multivariate parametric statistics with random-effects across subjects and sample covariance estimation across multiple measurements. Inferences were performed at the level of individual clusters (groups of contiguous voxels). Cluster-level inferences were based on parametric statistics from Gaussian Random Field theory ([Worsley et al., 1996](#_ENREF_29)). Results were thresholded using a combination of a cluster-forming p < 0.001 voxel-level threshold, and a familywise corrected p-FDR < 0.05 cluster-size threshold ([Chumbley et al., 2010](#_ENREF_9)).

**Clinical symptom and trait assessments**

In this study, we used a retrospectively acquired neuroimaging dataset. The authors of the CNP cohort provided details in support of the lifetime primary diagnosis described in our study, whereby 81% of the patients had at least one comorbidity. Table 1 summarizes the demographic and clinical assessment group descriptive statistics for the participants included in this study from the CNP dataset. The authors designate diagnoses according to the DSM-IV TR criteria ([American Psychiatric Association, 2000](#_ENREF_1)) through the Structured Clinical Interview for DSM-IV and SCID-I ([American Psychiatric Association, 2000](#_ENREF_1)) and complemented by the Adult ADHD Interview to better characterize a lifetime history of ADHD in adults ([Kaufman et al., 2000](#_ENREF_17)). In this study, 5 different behavioral assessments were compiled for symptoms and traits for each subject—the Adult ADHD Self-Report Scale v1.1 (ASRS) score; the Hopkins Symptom Checklist (HSCL with 6 subscales) for interpersonal sensitivity, somatization, anxiety, obsessive-compulsion, depression, and a global severity score; Dickman functional and dysfunctional impulsivity scales (total, positive, and negative for combined 6 subscales); Chapman Scales for Anhedonia (physical and social subscales); and the Scale for Traits that Increase Risk for Bipolar II Disorder.

While the authors provided data for several other assessments taken from the same patients, we chose these available assessments for several reasons. We wanted to obtain assessments that included healthy control subjects. Second, we wanted to determine whether these measures were associated with significant ROI pairs in both the task and task-free conditions. These clinical assessments were either conducted by a clinician or self-assessed by the subject. Additionally, we chose assessments that may also be more sensitive in declarative encoding and retrieval memory tasks.

The ASRS is a self-report screening tool used to evaluate whether a subject has symptoms of ADHD and was developed in conjunction with the World Health Organization (WHO), where the questions are consistent with DSM-IV criteria and address manifestations of ADHD symptoms in adults. The content of the questionnaire also reflects the importance that the DSM-IV places on symptoms, impairments, and history for a correct diagnosis ([American Psychiatric Association, 2000](#_ENREF_1)).

The HSCL is a self-reported inventory of 58 items representative of symptom configurations commonly observed in outpatients ([Derogatis et al., 1974](#_ENREF_10)). After consulting 58 items, the HSCL is scored on five underlying symptom dimensions—somatization, obsessive-compulsion, interpersonal sensitivity, anxiety, and depression.

The Dickman functional and dysfunctional impulsivity scales are 63-item self-reports used to assess separable components of impulsivity ([Dickman, 1990](#_ENREF_11)). Impulsivity is significantly correlated with individuals at greater risk of substance abuse and furthered with individuals with greater dysfunctional impulsivity. Dysfunctional impulsivity is associated with both disruptive behaviors and a tendency to ignore hard facts before deciding. However, functional impulsivity is the tendency to make quick decisions when such decisions are appropriate for the situation at hand. Overall, functional impulsivity has been associated with improved executive functioning. We anticipated that during paired-association memory tasks, there was a greater association with impulsivity scores.

The Chapman scale comprises 40 items for physical anhedonia and 48 items for social anhedonia to assess the decreased ability to experience pleasure ([Chapman et al., 1976](#_ENREF_8)). Physical anhedonia is a measure of the inability to experience pleasure from physical stimuli. Social anhedonia is a measure of social anxiety or avoidant behavior. Originally, this assessment was developed to classify certain groups of schizophrenic patients experiencing physical and/or social anhedonia.

The Scale for Traits that Increases Risk for Bipolar II Disorder used in the original study included subscales associated with social anxiety, energy level, and mood liability. While these traits are associated with bipolar II disorder, there are likely other traits that may be included in the other assessment tools, such as impulsivity ([Swann et al., 2009](#_ENREF_26)).

**Functional-based atlases**

In this study, we sought to demonstrate that shared functional connectivity patterns are present across these three disorders, both in cognitive tasks and task-free conditions. Our study incorporates three functional-based parcellation schemes to define our regions of interest within the cerebellum ([Ren et al., 2019](#_ENREF_22)), cerebral cortex ([Schaefer et al., 2018](#_ENREF_24)), and subcortex ([Tian et al., 2020](#_ENREF_27)). These atlases leverage functional connectivity gradients to determine the probabilistic organizational topography of anatomical parcels that go beyond typical histological atlases; in particular, task-evoked fMRI reveals a subtle organizational change in response to changing cognitive demands ([Tian et al., 2020](#_ENREF_27)). These features in our a priori atlas choices resemble our experimental system and should strengthen our explanatory predictions.

With the drastic increase in available functional neuroimaging methods and quantitative approaches for analyzing dynamic functional network organization within the human brain, choosing the appropriate regional brain atlases from which functional dynamics are inferred is crucial. The parcellation scale, shape, anatomical topology, network topology, and structural function correlation may change the hypothesis testing power as a result of atlas choice and atlas features ([Revell et al., 2022](#_ENREF_23)). This poses a challenge for the reproducibility and interpretation of underlying disease pathophysiology compared to healthy subjects. The choice of underlying brain atlas should follow good descriptive, explanatory, and predictive validity in the context of the experiment and hypothesis being tested ([Revell et al., 2022](#_ENREF_23)).

**Functional Network Connectivity (FNC)**

In this study, we used a cluster-level statistical inference based on multivariate statistics in ROI-to-ROI analysis using a second-level between-group general linear model. This procedure produced a single statistical matrix of F values, characterizing the effect of interest among all possible pairs in a network of 115 ROIs from 354 available ROIs. Three functional-based parcellation schemes were used to define 45 ROIs (out of 100, bilaterally comprising overlapping regions of Lobule VIIb, Vermis, Crus I, and Crus II) within the cerebellum ([Ren et al., 2019](#_ENREF_22)), 16 ROIs within the cerebral cortex ([Schaefer et al., 2018](#_ENREF_24)) (bilaterally comprising cortical regions of the default and salience networks), and all 54 bilateral ROIs within the subcortex ([Tian et al., 2020](#_ENREF_27)). FNC ([Jafri et al., 2008](#_ENREF_16)) uses a hierarchical clustering procedure based on region of interest (ROI)-to-region of interest (ROI) anatomical proximity and functional similarity metrics (Step 1, Supplemental Figure 1). Once these networks of ROIs are defined, FNC analyzes the entire set of connections between all pairs of ROIs in terms of the within- and between-network connectivity sets (Step 2, Supplemental Figure 1). The final step was to perform a multivariate parametric GLM analysis for all connections included in each of these sets/clusters of connections. In our study, we report the F-statistic for each pair found within the cluster of derived subnetworks and the associated p value as the false detection rate. This was defined as the expected portion of false discoveries among all pairs of networks with similar or larger effects across the entire set of FNC pairs.

**
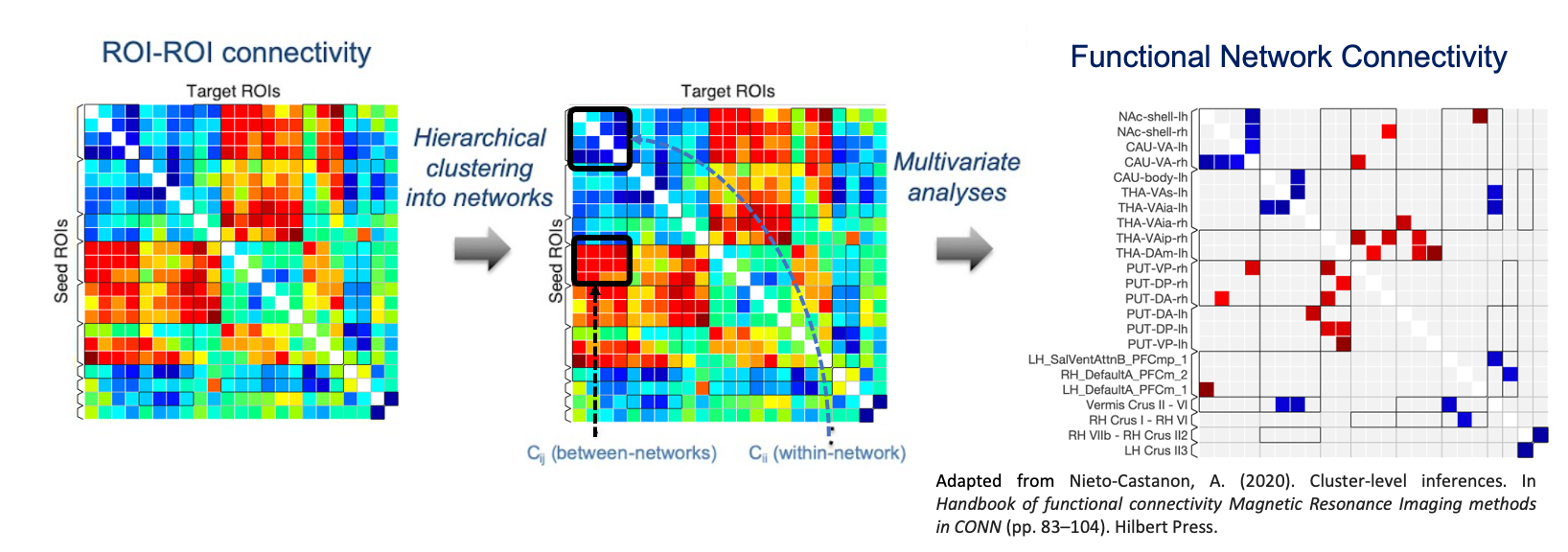
**

**Supplemental Figure 1: Functional network connectivity (FNC) cluster-level analysis method.**

Hierarchical clustering based upon ROI-to-ROI anatomical proximity and functional similarity metrics (all ROI pairs examined for within- and between-intrinsic network connectivity) was utilized to yield functional network connectivity (FNC) sets that survived multivariate parametric general linear model testing and familywise error control with the connection threshold set at p < 0.05 FDR-corrected and the cluster threshold set at p < 0.05 false discovery rate (FDR). Example: task-free SCHZ > HV [n=66, T(64)] ([Jafri et al., 2008](#_ENREF_16)).

**References**

American Psychiatric Association. (2000). *Diagnostic and statistical manual of mental disorders* (4th edn., text revision (DSM-IV-TR) ed.).

Andersson, J. L., Hutton, C., Ashburner, J., Turner, R., & Friston, K. (2001). Modeling geometric deformations in EPI time series. *Neuroimage*, *13*(5), 903-919. <https://doi.org/10.1006/nimg.2001.0746>

Ashburner, J. (2007). A fast diffeomorphic image registration algorithm. *Neuroimage*, *38*(1), 95-113. <https://doi.org/10.1016/j.neuroimage.2007.07.007>

Ashburner, J., & Friston, K. J. (2005). Unified segmentation. *Neuroimage*, *26*(3), 839-851. <https://doi.org/10.1016/j.neuroimage.2005.02.018>

Behzadi, Y., Restom, K., Liau, J., & Liu, T. T. (2007). A component based noise correction method (CompCor) for BOLD and perfusion based fMRI. *Neuroimage*, *37*(1), 90-101. <https://doi.org/10.1016/j.neuroimage.2007.04.042>

Calhoun, V. D., Wager, T. D., Krishnan, A., Rosch, K. S., Seymour, K. E., Nebel, M. B., Mostofsky, S. H., Nyalakanai, P., & Kiehl, K. (2017). The impact of T1 versus EPI spatial normalization templates for fMRI data analyses. *Hum Brain Mapp*, *38*(11), 5331-5342. <https://doi.org/10.1002/hbm.23737>

Chai, X. J., Castanon, A. N., Ongur, D., & Whitfield-Gabrieli, S. (2012). Anticorrelations in resting state networks without global signal regression. *Neuroimage*, *59*(2), 1420-1428. <https://doi.org/10.1016/j.neuroimage.2011.08.048>

Chapman, L. J., Chapman, J. P., & Raulin, M. L. (1976). Scales for physical and social anhedonia. *J Abnorm Psychol*, *85*(4), 374-382. <https://doi.org/10.1037//0021-843x.85.4.374>

Chumbley, J., Worsley, K., Flandin, G., & Friston, K. (2010). Topological FDR for neuroimaging. *Neuroimage*, *49*(4), 3057-3064. <https://doi.org/10.1016/j.neuroimage.2009.10.090>

Derogatis, L. R., Lipman, R. S., Rickels, K., Uhlenhuth, E. H., & Covi, L. (1974). The Hopkins Symptom Checklist (HSCL): a self-report symptom inventory. *Behav Sci*, *19*(1), 1-15. <https://doi.org/10.1002/bs.3830190102>

Dickman, S. J. (1990). Functional and dysfunctional impulsivity: personality and cognitive correlates. *J Pers Soc Psychol*, *58*(1), 95-102. <https://doi.org/10.1037//0022-3514.58.1.95>

Friston, K. J., Ashburner, J., Frith, C. D., Poline, J. B., Heather, J. D., & Frackowiak, R. S. J. (2004). Spatial registration and normalization of images. *Human Brain Mapping*, *3*(3), 165-189. <https://doi.org/10.1002/hbm.460030303>

Friston, K. J., Buechel, C., Fink, G. R., Morris, J., Rolls, E., & Dolan, R. J. (1997). Psychophysiological and modulatory interactions in neuroimaging. *Neuroimage*, *6*(3), 218-229. <https://doi.org/10.1006/nimg.1997.0291>

Friston, K. J., Williams, S., Howard, R., Frackowiak, R. S., & Turner, R. (1996). Movement-related effects in fMRI time-series. *Magn Reson Med*, *35*(3), 346-355. <https://doi.org/10.1002/mrm.1910350312>

Hallquist, M. N., Hwang, K., & Luna, B. (2013). The nuisance of nuisance regression: spectral misspecification in a common approach to resting-state fMRI preprocessing reintroduces noise and obscures functional connectivity. *Neuroimage*, *82*, 208-225. <https://doi.org/10.1016/j.neuroimage.2013.05.116>

Jafri, M. J., Pearlson, G. D., Stevens, M., & Calhoun, V. D. (2008). A method for functional network connectivity among spatially independent resting-state components in schizophrenia. *Neuroimage*, *39*(4), 1666-1681. <https://doi.org/10.1016/j.neuroimage.2007.11.001>

Kaufman, J., Birmaher, B., Brent, D. A., Ryan, N. D., & Rao, U. (2000). K-Sads-Pl. *J Am Acad Child Adolesc Psychiatry*, *39*(10), 1208. <https://doi.org/10.1097/00004583-200010000-00002>

McLaren, D. G., Ries, M. L., Xu, G., & Johnson, S. C. (2012). A generalized form of context-dependent psychophysiological interactions (gPPI): a comparison to standard approaches. *Neuroimage*, *61*(4), 1277-1286. <https://doi.org/10.1016/j.neuroimage.2012.03.068>

Morfini, F., Whitfield-Gabrieli, S., & Nieto-Castanon, A. (2023). Functional connectivity MRI quality control procedures in CONN. *Front Neurosci*, *17*, 1092125. <https://doi.org/10.3389/fnins.2023.1092125>

Penny, W. F., K.; Ashburner, J.; Kiebel, S.; Nichols, T. (2011). *Statistical Parametric Mapping: The Analysis of Functional Brain Images*. Academic Press.

Power, J. D., Mitra, A., Laumann, T. O., Snyder, A. Z., Schlaggar, B. L., & Petersen, S. E. (2014). Methods to detect, characterize, and remove motion artifact in resting state fMRI. *Neuroimage*, *84*, 320-341. <https://doi.org/10.1016/j.neuroimage.2013.08.048>

Ren, Y., Guo, L., & Guo, C. C. (2019). A connectivity-based parcellation improved functional representation of the human cerebellum. *Sci Rep*, *9*(1), 9115. <https://doi.org/10.1038/s41598-019-45670-6>

Revell, A. Y., Silva, A. B., Arnold, T. C., Stein, J. M., Das, S. R., Shinohara, R. T., Bassett, D. S., Litt, B., & Davis, K. A. (2022). A framework For brain atlases: Lessons from seizure dynamics. *Neuroimage*, *254*, 118986. <https://doi.org/10.1016/j.neuroimage.2022.118986>

Schaefer, A., Kong, R., Gordon, E. M., Laumann, T. O., Zuo, X. N., Holmes, A. J., Eickhoff, S. B., & Yeo, B. T. T. (2018). Local-Global Parcellation of the Human Cerebral Cortex from Intrinsic Functional Connectivity MRI. *Cereb Cortex*, *28*(9), 3095-3114. <https://doi.org/10.1093/cercor/bhx179>

Sladky, R., Friston, K. J., Trostl, J., Cunnington, R., Moser, E., & Windischberger, C. (2011). Slice-timing effects and their correction in functional MRI. *Neuroimage*, *58*(2), 588-594. <https://doi.org/10.1016/j.neuroimage.2011.06.078>

Swann, A. C., Lijffijt, M., Lane, S. D., Steinberg, J. L., & Moeller, F. G. (2009). Increased trait-like impulsivity and course of illness in bipolar disorder. *Bipolar Disord*, *11*(3), 280-288. <https://doi.org/10.1111/j.1399-5618.2009.00678.x>

Tian, Y., Margulies, D. S., Breakspear, M., & Zalesky, A. (2020). Topographic organization of the human subcortex unveiled with functional connectivity gradients. *Nat Neurosci*, *23*(11), 1421-1432. <https://doi.org/10.1038/s41593-020-00711-6>

Whitfield-Gabrieli, S., & Nieto-Castanon, A. (2012). Conn: a functional connectivity toolbox for correlated and anticorrelated brain networks. *Brain Connect*, *2*(3), 125-141. <https://doi.org/10.1089/brain.2012.0073>

Worsley, K. J., Marrett, S., Neelin, P., Vandal, A. C., Friston, K. J., & Evans, A. C. (1996). A unified statistical approach for determining significant signals in images of cerebral activation. *Human Brain Mapping*, *4*(1), 58-73. <https://doi.org/10.1002/(sici)1097-0193(1996)4:1><58::Aid-hbm4>3.0.Co;2-o
